# Governing AI-driven digital transformation in public healthcare: assessing administrative readiness and institutional capacity in Egypt's health system

**DOI:** 10.3389/fdgth.2026.1883794

**Published:** 2026-09-03

**Authors:** Mohamed Abbas El-Naggar, Dina Helmy Al Nashily, Hiyam Ahmed Abdulrahim, Abdelrehim Awad, Zahid Hussain, Anber Abraheem Shlash Mohammad

**Affiliations:** 1 Department of General Subjects, University of Business and Technology, Jeddah, Saudi Arabia; 2 Chemical Engineering Department, Faculty of Engineering, Alexandria University, Alexandria, Egypt; 3 Department of Business Administration, Faculty of Commerce, Al-Azhar University, Cairo, Egypt; 4 Department of Economics, College of Business Administration, Princess Nourah bint Abdulrahman University, Riyadh, Saudi Arabia; 5 Department of Business Administration, College of Business, University of Bisha, Bisha, Saudi Arabia; 6 Department of Business Administration, KASBIT, Karachi, Pakistan; 7 Digital Marketing Department, Faculty of Administrative and Financial Sciences, University of Petra, Petra, Jordan

**Keywords:** administrative readiness, artificial intelligence governance, digital health transformation, Egypt, health systems, institutional capacity, public healthcare

## Abstract

**Background:**

Artificial intelligence (AI) and digital health technologies are increasingly shaping how public healthcare services are planned, delivered, and governed. In Egypt, national digital health reforms have created an urgent need to assess whether public healthcare institutions have the administrative readiness, institutional capacity, and governance arrangements required for AI-enabled digital health transformation.

**Methods:**

A sequential explanatory mixed-methods design was employed across 24 Egyptian governorates. Phase 1 consisted of a cross-sectional survey of 387 healthcare administrators and policymakers from Ministry of Health and Population hospitals, university hospitals, primary healthcare units, and Health Insurance Organization facilities. The survey measured eight operationalized constructs: institutional capacity, administrative readiness, organizational governance, leadership support, IT infrastructure, staff training and skills, regulatory framework, and digital health transformation success. Phase 2 comprised semi-structured interviews with 18 senior policymakers, hospital leaders, digital health consultants, health information system managers, and health policy researchers. Quantitative findings were analyzed using descriptive statistics, confirmatory factor analysis, and structural equation modeling, while qualitative data were analyzed thematically and integrated with the survey results through explanatory joint display logic.

**Results:**

Among survey respondents, 62.3% were male, 71.6% held a master's degree or higher, and 58.1% had more than 10 years of professional experience. The structural model showed acceptable fit (CMIN/DF = 2.14, CFI = .96, TLI = .95, RMSEA = .045, SRMR = .038) and explained 68% of the variance in perceived digital health transformation success. Institutional capacity, administrative readiness, and organizational governance were positively associated with transformation success. The specified indirect pathways through leadership support, IT infrastructure, staff training and skills, and regulatory framework were also significant and are interpreted as hypothesis-generating associations because of the cross-sectional design. Interview findings helped explain the quantitative patterns by identifying fragmented governance, weak infrastructure, workforce digital literacy gaps, regulatory ambiguity, and resource allocation constraints.

**Conclusions:**

Egypt's public healthcare system faces interrelated institutional, administrative, workforce, infrastructure, and regulatory barriers to AI-enabled digital health transformation. Strengthening governance coordination, infrastructure equity, workforce capability, and AI-specific regulatory safeguards is essential before large-scale AI deployment can be reliably translated into public value.

## 1 Introduction

The study objectives were revised for clarity as follows: (1) to describe current levels of institutional capacity and administrative readiness for AI-enabled digital health transformation in Egyptian public healthcare institutions; (2) to operationalize and assess the key constructs that constitute digital health readiness and governance capacity; (3) to examine associations among institutional capacity, administrative readiness, organizational governance, mediating readiness mechanisms, and perceived digital health transformation success; and (4) to use qualitative interviews to explain the quantitative results and identify governance challenges and policy priorities.

Despite the growing literature on digital health transformation, three gaps remain. First, many studies emphasize technology adoption while giving less attention to administrative and institutional readiness. Second, evidence on AI governance remains concentrated in high-income health systems, with limited empirical work from low- and middle-income countries and the MENA region. Third, studies on digital health readiness in Egypt are often descriptive and provide limited evidence on how readiness factors are associated with perceived transformation outcomes (1–5). Therefore, this study develops and tests an integrated readiness model for AI-enabled digital health transformation in Egypt's public healthcare system.

Organizational governance forms the third pillar of the framework. In healthcare, governance refers to the structures, norms, rules, accountability mechanisms, and stakeholder-participation processes through which decisions are made and implemented (6–8). For AI-enabled digital health transformation, governance must also address algorithmic accountability, data provenance, model validation, bias monitoring, patient safety, privacy protection, and post-deployment surveillance (9–12).

Administrative readiness is treated as a related but distinct pillar. It refers to the preparedness of managerial systems, operational processes, staff competencies, decision routines, and internal coordination mechanisms to support the adoption and governance of digital health technologies (3, 13, 14). Prior work in Egypt and comparable low- and middle-income contexts suggests that weak strategic planning, limited performance management, fragmented coordination, and uneven digital skills often constrain health system reform (1, 2, 4, 15). These administrative constraints are intensified by the rapid pace of technological change and by the difficulty public institutions face in updating rules, workflows, and workforce capabilities at the same speed as digital innovation.

This policy ambition raises a practical governance question: are public healthcare institutions sufficiently prepared to adopt and govern AI-enabled digital health transformation? In this study, institutional capacity is defined as the availability of organizational mandates, human resources, financial resources, technical capability, coordination mechanisms, and administrative routines that allow public healthcare institutions to absorb and sustain digital transformation. This definition distinguishes institutional capacity from technology procurement alone and links it to the ability of organizations to implement, regulate, and maintain innovation over time (16–19).

Egypt provides an important setting for examining these issues because it has a complex public healthcare system that includes Ministry of Health and Population facilities, Health Insurance Organization facilities, university hospitals, military healthcare facilities, and a growing private sector (20–22). National health reform priorities increasingly emphasize interoperable digital platforms, health information infrastructure, universal health coverage, and the responsible integration of advanced analytics into healthcare delivery and governance.

Artificial intelligence (AI) and digital health technologies are transforming public healthcare governance by expanding the use of predictive analytics, digital decision support, automated resource allocation, and data-driven service planning (23–25). The World Health Organization's guidance on ethics and governance of AI for health remains a foundational international reference and should be read alongside recent governance reports emphasizing implementation capacity, data stewardship, institutional accountability, and post-deployment oversight (26, 27).

## 2 Materials and methods

### 2.1 Study design and setting

This study used a sequential explanatory mixed-methods design (28). The quantitative strand was conducted first through a cross-sectional survey, followed by a qualitative interview strand designed to explain and contextualize the survey findings. The two strands were integrated at interpretation through a joint-display approach that connected statistical patterns with interview themes. This structure was adopted to make the mixing explicit: the survey identified the relative strength of readiness and governance associations, while the interviews explained why those patterns occurred in practice.

The setting comprised public healthcare institutions in 24 of Egypt's 27 governorates, covering metropolitan, urban, peri-urban, and rural/Upper Egypt contexts. Institutions included Ministry of Health and Population hospitals, university hospitals, primary healthcare units, and Health Insurance Organization facilities. The study period extended from September 2025 to January 2026. No clinical intervention, patient treatment, or identifiable patient record extraction was conducted.

### 2.2 Theoretical framework

**Figure 1:**
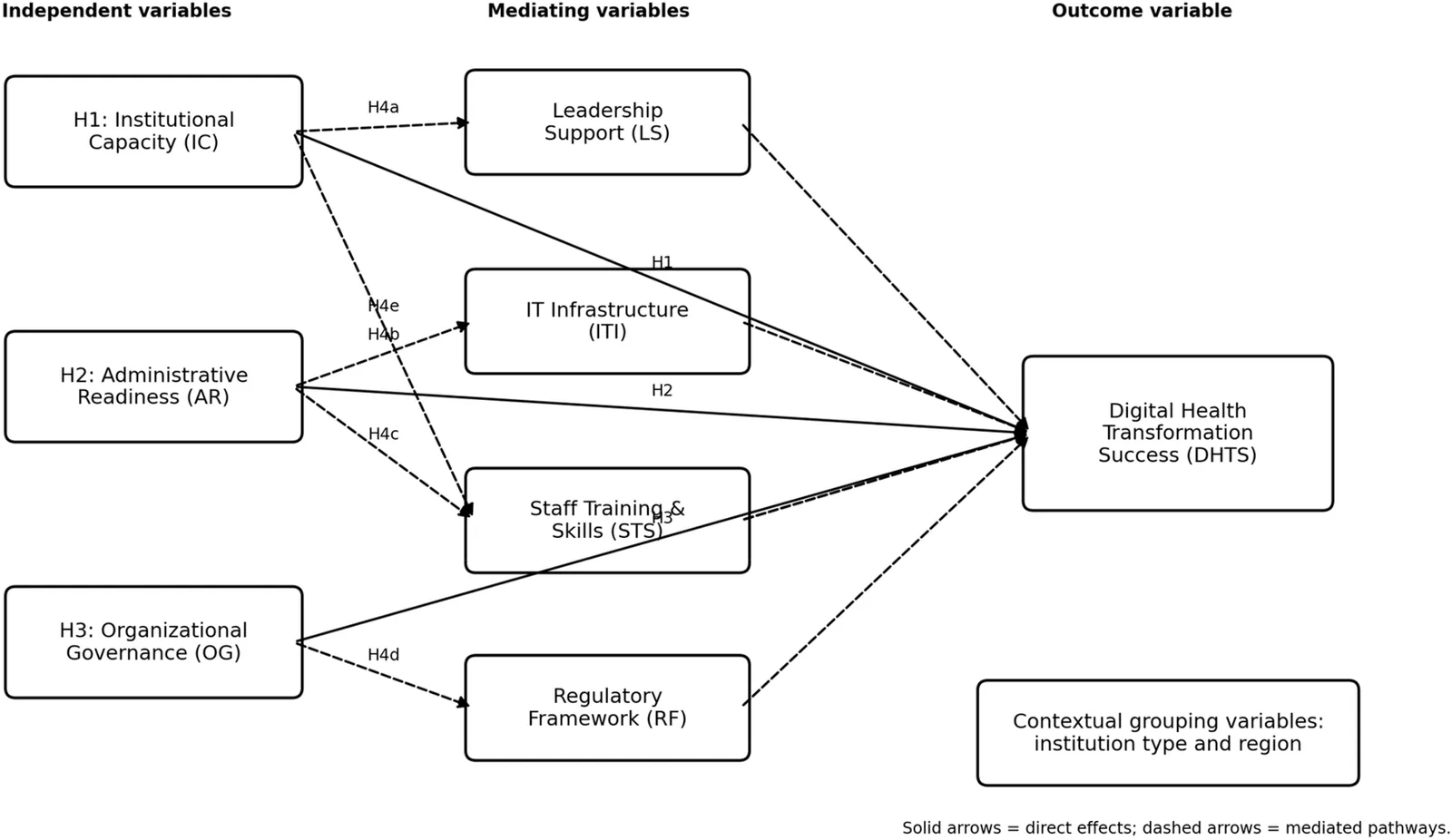
Conceptual framework.

The conceptual framework (Figure 1) was developed through three steps. First, the authors reviewed institutional capacity scholarship, technology-acceptance theory, health-system governance frameworks, and recent AI-health governance evidence (7, 9, 18, 26, 27, 29). Second, constructs were mapped to the Egyptian digital health context through expert review. Third, the model was refined to include three exogenous readiness pillars—institutional capacity, administrative readiness, and organizational governance—and four specified mediating mechanisms: leadership support, IT infrastructure, staff training and skills, and regulatory framework. The outcome variable was digital health transformation success. Contextual grouping variables, institution type and geographic region, were examined descriptively rather than as formal moderators because measurement invariance testing was not performed.

Figure 1 showing the hypothesized direct effects of institutional capacity, administrative readiness, and organizational governance on digital health transformation success, and the specified mediating pathways through leadership support, IT infrastructure, staff training and skills, and regulatory framework.

### 2.3 Study population and sampling

#### 2.3.1 Quantitative survey

The target respondents were healthcare administrators, policymakers, and operational managers involved in planning, governing, or implementing digital health activities in public healthcare institutions. Eligible participants included hospital directors, deputy directors, heads of medical departments, IT managers, quality directors, assurance officers, and health policy planners. Because no single national registry of eligible administrators across all selected institutions was available to the authors, the sampling frame was constructed from institutional staff lists provided by participating facilities. The lack of a complete national denominator is acknowledged as a sampling limitation.

Governorates were stratified into metropolitan, urban, and rural/Upper Egypt groups. Facilities were then selected within each stratum, and respondents were invited from relevant administrative and technical roles. The sampling strategy was designed to ensure geographic and institutional diversity rather than to produce nationally weighted estimates. Subgroup comparisons by institution type and region were therefore treated as exploratory.

The minimum sample size was calculated using the Krejcie and Morgan (30) formula for finite populations and was cross-checked against accepted structural equation modeling guidance (31). A minimum of 350 completed responses was required to achieve 0.80 statistical power at alpha = .05 for medium standardized path coefficients. The final realized sample of 387 valid responses exceeded this requirement. Details of questionnaires distributed, returned, and excluded are reported in the Results section as sample realization.

#### 2.3.2 Qualitative interviews

After preliminary survey analysis, 18 participants were purposively selected for semi-structured interviews. Selection aimed for maximum variation by institution type, professional role, geographic location, and experience with digital health governance. Interviewees included senior Ministry of Health and Population officials (*n* = 4), university hospital directors (*n* = 3), Health Insurance Organization representatives (*n* = 2), digital health consultants working with international organizations in Egypt (*n* = 3), health information systems managers (*n* = 3), and health policy researchers (*n* = 3).

### 2.4 Data collection instruments

The quantitative survey instrument was a structured, self-administered questionnaire consisting of 68 items distributed across eight operationalized constructs. Table 1 defines each construct, indicates the number of items, and provides examples of the AI-relevant governance content captured by the instrument. This revision clarifies that the former wording referring to “CT support” was erroneous and has been removed.

**Table 1:** Operational definitions and measurement structure of the survey constructs.

Construct	Operational definition	Items	Example measurement content
Institutional capacity	Organizational resources, mandates, coordination arrangements, and technical-administrative capability available to support digital health and AI governance.	9	Budget availability; interdepartmental coordination; technical resources; ability to support AI-enabled tools.
Administrative readiness	Preparedness of managerial processes, workflows, planning routines, and staff procedures for technology-enabled health transformation.	9	Strategic planning; process readiness; workflow flexibility; readiness to adopt AI-supported administrative systems.
Organizational governance	Accountability, transparency, stakeholder participation, decision authority, and oversight structures for digital health initiatives.	8	Clear governance responsibilities; accountability for technology decisions; transparent decision-making.
Leadership support	Visible commitment, resource allocation, and change-management support from senior leaders.	8	Leadership sponsorship; support for staff adoption; resource commitment for digital health.
IT infrastructure	Availability, interoperability, reliability, connectivity, cybersecurity, and data-management capacity of digital systems.	8	Interoperability; network reliability; data infrastructure; readiness for AI-enabled analytics.
Staff training and skills	Digital literacy, health informatics competence, and AI-related capability among administrative and technical staff.	8	Training availability; digital competencies; understanding of AI-related risks and uses.
Regulatory framework	Formal rules, standards, and safeguards governing digital health and AI use.	8	Data provenance; algorithmic accountability; model validation expectations; bias monitoring; privacy and patient-safety safeguards.
Digital health transformation success	Perceived progress in achieving service, data, efficiency, and governance outcomes from digital health transformation.	10	Service delivery efficiency; data integration; cost-effectiveness; readiness for AI-enabled decision support.

All items were rated on a 5-point Likert scale (1 = strongly disagree to 5 = strongly agree). Items were adapted from established literature on institutional capacity, organizational readiness, governance, technology acceptance, and digital transformation capability (7, 14, 17, 18, 29). AI-specific governance content was incorporated particularly into the regulatory framework, IT infrastructure, staff training, and transformation-success constructs to cover algorithmic accountability, data provenance, model validation, bias monitoring, post-deployment oversight, and privacy safeguards (9, 10, 12, 26). The instrument was translated and back-translated according to WHO translation and adaptation guidance (32).

Content validity was assessed before full data collection by a panel of eight experts in public health, public administration, health informatics, and digital governance from four Egyptian universities. Experts evaluated item relevance, clarity, and contextual suitability. Individual item content-validity indices ranged from 0.83 to 1.00, and the scale-level CVI average was 0.94, exceeding the recommended threshold of 0.80 (33). Construct validity and reliability were subsequently examined through confirmatory factor analysis, composite reliability, average variance extracted, and discriminant-validity checks.

The qualitative interview guide was aligned with the survey model and covered five areas: (a) digital health infrastructure; (b) governance challenges for AI adoption; (c) workforce capacity and training needs; (d) regulatory and policy environment; and (e) priorities for strengthening AI governance. Interviews lasted 45–75 min, were conducted in Arabic, audio-recorded with consent, transcribed verbatim, and anonymized before analysis.

### 2.5 Data analysis

Survey data were analyzed using SPSS 28 and AMOS 28. Descriptive statistics were used to address the first two objectives by summarizing current levels of institutional capacity, administrative readiness, governance, and related readiness constructs. Pearson correlations were used to examine bivariate associations. The hypothesized model was tested using structural equation modeling with maximum likelihood estimation. Model fit was evaluated using CMIN/DF, CFI, TLI, RMSEA, and SRMR following established SEM guidance (31). Bootstrapping with 5,000 iterations was used to estimate bias-corrected 95% confidence intervals for the specified indirect associations.

Because the study was cross-sectional, the SEM paths and indirect pathways were interpreted as associations rather than causal effects. Multicollinearity and construct overlap were examined using the correlation matrix, discriminant-validity evidence, and collinearity diagnostics. Correlations below.85 and acceptable discriminant-validity statistics supported retention of the constructs as related but distinct dimensions of readiness. Common method bias was addressed procedurally through anonymity, neutral wording, and separation of construct blocks within the questionnaire, and statistically through a CFA-based common latent factor sensitivity check rather than relying solely on Harman's single-factor test.

Interview data were analyzed using Braun and Clarke's thematic analysis approach (34, 35). Transcripts were imported into NVivo 14 and coded inductively and deductively. Trustworthiness was strengthened through member checking, peer debriefing by two independent researchers, and thick description. Mixed-methods integration was conducted after separate quantitative and qualitative analyses. The qualitative themes were used to explain quantitative findings through convergence, expansion, and contextualization; the integrated interpretation is presented in the Results and Discussion.

## 3 Results

### 3.1 Participant characteristics

1Sample realization. A total of 520 questionnaires were distributed, 412 were returned (response rate = 79.2%), and 387 were retained after excluding incomplete responses and questionnaires that failed attention checks. Table 2 summarizes the demographic and organizational characteristics of the 387 valid survey respondents. The majority were male (62.3%), held a master's degree or higher (71.6%), and had more than 10 years of professional experience in the health sector (58.1%). Respondents were distributed across MoHP hospitals (42.4%), university hospitals (24.8%), primary healthcare facilities (18.9%), and Health Insurance Organization facilities (13.9%). Geographic representation included metropolitan areas (38.5%), urban governorates (31.5%), and rural/Upper Egypt governorates (30.0%).

**Table 2:** Demographic and organizational characteristics of survey respondents (*N* = 387).

Characteristic	Category	*n* (%)
Gender	Male	241 (62.3)
	Female	146 (37.7)
Age group	30–39 years	68 (17.6)
	40–49 years	148 (38.2)
	50–59 years	132 (34.1)
	60+ years	39 (10.1)
Education level	Bachelor's degree	110 (28.4)
	Master's degree	172 (44.4)
	Doctoral degree (PhD/MD)	105 (27.2)
Experience	<10 years	162 (41.9)
	10–20 years	148 (38.2)
	>20 years	77 (19.9)
Institution type	MoHP hospital	164 (42.4)
	University hospital	96 (24.8)
	Primary healthcare unit	73 (18.9)
	Health Insurance Org.	54 (13.9)
Region	Metropolitan	149 (38.5)
	Urban	122 (31.5)
	Rural/Upper Egypt	116 (30.0)
Position level	Senior management	84 (21.7)
	Middle management	186 (48.1)
	Operational level	117 (30.2)

### 3.2 Descriptive statistics and correlations

The descriptive statistics and inter-construct correlations are shown in Table 3. Mean scores for institutional capacity (*M* = 2.87, SD = 0.78), administrative readiness (*M* = 2.74, SD = 0.81), and organizational governance (*M* = 2.92, SD = 0.72) were below the scale midpoint, suggesting low-to-moderate readiness. Digital health transformation success had the lowest mean (*M* = 2.51, SD = 0.86). Leadership support had the highest mean (*M* = 2.96, SD = 0.75), whereas the regulatory framework had the lowest among the enabling mechanisms (*M* = 2.58, SD = 0.82). All inter-construct correlations were positive and statistically significant (*p* < .01).

**Table 3:** Descriptive statistics and Pearson correlations among study variables (*N* = 387).

Variable	*M*	SD	1	2	3	4	5	6	7	8
1. IC	2.87	0.78	—							
2. AR	2.74	0.81	.68**	—						
3. OG	2.92	0.72	.63**	.59**	—					
4. LS	2.96	0.75	.65**	.61**	.72**	—				
5. ITI	2.64	0.84	.58**	.55**	.52**	.60**	—			
6. STS	2.61	0.79	.54**	.51**	.48**	.56**	.62**	—		
7. RF	2.58	0.82	.50**	.47**	.49**	.53**	.45**	.41**	—	
8. DHTS	2.51	0.86	.64**	.61**	.58**	.67**	.70**	.63**	.55**	—

M, mean; SD, standard deviation. IC, institutional capacity; AR, administrative readiness; OG, organizational governance; LS, leadership support; ITI, IT infrastructure; STS, staff training and skills; RF, regulatory framework; DHTS, digital health transformation success.

** *p* < .01 (two-tailed).

The inter-construct correlations ranged from.41 to.72, indicating moderate to strong positive associations. The highest correlation was between organizational governance and leadership support (*r* = .72), which is theoretically expected because leadership is a governance mechanism. However, no correlation approached the.85 threshold commonly associated with serious multicollinearity, and discriminant-validity checks supported the constructs as related but distinct readiness dimensions.

### 3.3 Structural equation modeling results

The hypothesized structural model demonstrated acceptable fit to the data: CMIN/DF = 2.14, CFI = .96, TLI = .95, RMSEA = .045 (90% CI: .032–.058), SRMR = .038, and PCLOSE = .72. The model explained 68% of the variance in perceived digital health transformation success. Because of the cross-sectional design, the paths are interpreted as standardized associations rather than causal effects.

**Figure 2:**
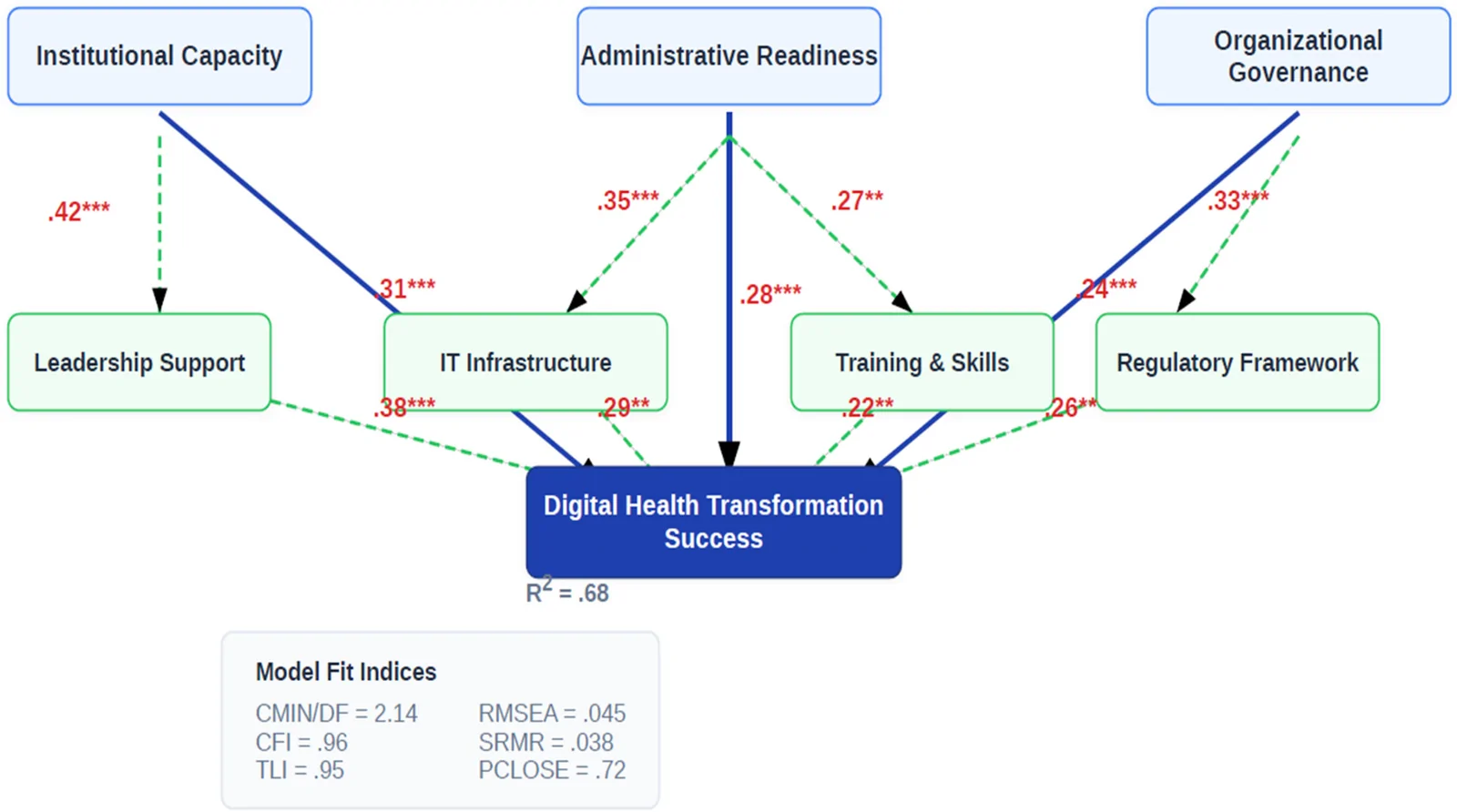
Structural equation modeling (SEM) path analysis results. Solid lines indicate significant direct associations; dashed lines indicate specified indirect pathways. Standardized path coefficients are shown; ***p < .001, **p < .01. The model explained 68% of the variance in perceived digital health transformation success.

The standardized path coefficients for the hypothesized associations are shown in Figure 2 and Table 4. Institutional capacity was positively associated with DHTS (*β* = .31, *p* < .001), administrative readiness was positively associated with DHTS (*β* = .28, *p* < .001), and organizational governance was positively associated with DHTS (*β* = .24, *p* < .001).

**Table 4:** Standardized path coefficients for the structural model (*N* = 387).

Hypothesis	Path	*β*	SE	Result
Direct effects				
H1	IC → DHTS	.31***	.04	Supported
H2	AR → DHTS	.28***	.04	Supported
H3	OG → DHTS	.24***	.04	Supported
Mediated effects				
H4a	IC → LS → DHTS	.42***	.05	Supported
H4b	AR → ITI → DHTS	.35***	.05	Supported
H4c	AR → STS → DHTS	.27**	.05	Supported
H4d	OG → RF → DHTS	.26**	.05	Supported
H4e	IC → STS → DHTS	.22**	.05	Supported
Model fit indices				
CMIN/DF = 2.14; CFI = .96; TLI = .95; RMSEA = .045; SRMR = .038; *R*^2^ (DHTS) = .68				

*β*, standardized coefficient; SE, standard error.

** *p* < .01.

*** *p* < .001.

The indirect-pathway analysis showed that only the specified pathways in Table 4 were tested. Leadership support showed the strongest indirect association for the IC-LS-DHTS pathway (*β* = .42, *p* < .001), followed by IT infrastructure for the AR-ITI-DHTS pathway (*β* = .35, *p* < .001). Staff training and skills and the regulatory framework also showed significant specified indirect associations. These mediation results are interpreted as hypothesis-generating evidence of plausible mechanisms rather than proof of causal mediation.

### 3.4 Subgroup comparisons by institution type and geographic region

Subgroup comparisons revealed significant differences across institutional types and geographic regions. Because measurement invariance testing and formal path-coefficient comparisons were not conducted, these analyses are reported as descriptive subgroup comparisons rather than full multi-group SEM. University hospitals demonstrated significantly higher levels of institutional capacity (*M* = 3.24 vs. 2.71 for MoHP hospitals, *p* < .001) and IT infrastructure (*M* = 3.12 vs. 2.43, *p* < .001). Metropolitan institutions scored significantly higher on all constructs compared to rural facilities, with the largest disparities observed in IT infrastructure (mean difference = 1.08, *p* < .001) and staff training (mean difference = 0.94, *p* < .001).

### 3.5 Qualitative findings

Before thematic interpretation, the qualitative sample was summarized to improve transparency. The 18 interview participants represented senior government, hospital, insurance, consulting, information-systems, and research perspectives. Thematic analysis then identified five major themes and 14 sub-themes (Table 5). The characteristics and professional backgrounds of the interview participants are summarized in Table 6.

**Table 5:** Summary of qualitative themes and sub-themes from semi-structured interviews (*N* = 18).

Theme	Sub-themes	Frequency
1. Fragmented governance structures	Inter-agency coordination gaps; overlapping mandates; lack of centralized AI oversight	16/18
2. Inadequate IT infrastructure	Outdated hardware/software; limited interoperability; connectivity gaps in rural areas	18/18
3. Workforce digital literacy gaps	Insufficient training programs; resistance to change; shortage of qualified IT staff	17/18
4. Regulatory ambiguity	Absence of AI-specific regulations; uncertainty for providers and vendors; concerns regarding patient safety, data privacy, and algorithmic bias	15/18
5. Resource allocation challenges	Insufficient IT budgets; dependency on donor funding; competing health priorities	14/18

**Table 6:** Characteristics of interview participants (*N* = 18).

Characteristic	Categories/*n*
Role group	MoHP officials (4); university hospital directors (3); Health Insurance Organization representatives (2); digital health consultants (3); health information systems managers (3); health policy researchers (3)
Institutional background	National public health administration; university hospitals; health insurance; digital health consulting; health information systems; policy research
Geographic perspective	Metropolitan, urban, and rural/Upper Egypt experience represented through professional roles
Selection rationale	Purposive maximum-variation sampling to explain survey results and identify governance challenges

#### 3.5.1 Theme 1: fragmented governance structures

Participants consistently highlighted the absence of a unified governance framework for digital health and AI adoption. As one senior MoHP official stated: “Each institution implements its own digital systems without coordination. We have multiple electronic health record systems that cannot communicate with each other. There is no single authority responsible for overseeing AI adoption in the health sector” (Participant 3, MoHP). This fragmentation was attributed to overlapping mandates among MoHP, the Cabinet's Information and Decision Support Center, the Ministry of Communications and Information Technology, university hospitals, and other public bodies. Participants explained that unclear accountability creates uncertainty about who should set AI standards, validate systems, monitor risks, and coordinate post-deployment oversight.

#### 3.5.2 Theme 2: inadequate IT infrastructure

All 18 participants cited infrastructure deficits as a central barrier. Hospital information technology managers referred to outdated operating systems, incompatible databases, limited interoperability, cybersecurity concerns, and insufficient bandwidth. Rural facilities were described as particularly disadvantaged, with unreliable connectivity and limited access to modern computing equipment.

#### 3.5.3 Theme 3: workforce digital literacy gaps

Seventeen participants identified insufficient digital literacy, limited health-informatics training, and a shortage of qualified IT staff as major barriers. Training programs were described as episodic rather than systematic and not yet aligned with the competencies required for AI-enabled healthcare governance, including data quality, algorithmic risk awareness, and responsible use of automated decision support.

#### 3.5.4 Theme 4: regulatory ambiguity

One key identified area of absence was the lack of AI-specific regulatory frameworks. Egypt has legislation on data protection (Personal Data Protection Law No. 151 of 2020) but no specific legislation on the use of AI in clinical decision making, diagnostic algorithms or health data analytics. This regulatory gap leaves patients, healthcare providers, and tech manufacturers uncertain, and with concerns for patient safety, data privacy, and algorithmic bias.

#### 3.5.5 Theme 5: resource allocation challenges

Financial constraints continued to limit digital transformation. Healthcare administrators noted that IT budgets are often underfunded and vulnerable to cuts during budgetary pressure. Participants also warned that donor-funded project-based digital health initiatives may not produce sustainable system-wide capacity unless they are embedded within long-term institutional funding and governance strategies.

#### 3.5.6 Mixed-methods integration

The qualitative findings explained the quantitative results in three ways. First, the low mean scores for administrative readiness and DHTS were consistent with interview accounts of weak coordination, limited infrastructure, and resource constraints. Second, the strong associations involving leadership support and IT infrastructure were explained by participants’ emphasis on leadership sponsorship and system interoperability as practical preconditions for transformation. Third, the regulatory framework pathway was supported by the qualitative theme of regulatory ambiguity, particularly concerns about algorithmic accountability, model validation, data privacy, and patient safety. This integration provides evidence of sequential mixing rather than two unrelated data strands.

## 4 Discussion

This study provides empirical and qualitative evidence on administrative and institutional readiness for AI-enabled digital health transformation in Egypt's public healthcare system. The findings should be interpreted as cross-sectional associations supplemented by explanatory qualitative evidence, not as definitive causal effects.

### 4.1 Key findings and theoretical contributions

The structural equation model accounted for 68% of the variance in perceived digital health transformation success, suggesting that institutional capacity, administrative readiness, and organizational governance are strongly associated with digital health readiness outcomes. This pattern supports the theoretical argument that digital transformation is shaped by institutional and organizational conditions, not by technology availability alone (18, 19, 27).

Institutional capacity showed the strongest standardized association with DHTS (*β* = .31). This is consistent with capacity-strengthening scholarship, which suggests that organizations with clearer mandates, adequate resources, capable human capital, and effective processes are better positioned to absorb complex innovations (16–18). The result should not be read as proof that institutional capacity causes transformation success, but as evidence that these constructs move together in the surveyed institutions.

Administrative readiness was also positively associated with DHTS (beta = .28). The low mean score for administrative readiness (*M* = 2.74) indicates that many institutions may lack the management processes, workflow flexibility, and workforce preparedness needed for AI-enabled digital health initiatives.

Organizational governance was positively associated with DHTS (*β* = .24) and was also linked to the regulatory framework and leadership mechanisms in the specified model. This pattern aligns with health-system governance principles and emerging AI-health governance literature emphasizing accountability, transparency, participation, safety, and rule-based oversight (7, 9, 11, 12, 26).

The indirect-pathway findings suggest that leadership support and IT infrastructure may be important mechanisms through which institutional and administrative readiness are translated into perceived transformation outcomes. These results are best understood as hypothesis-generating because the data are cross-sectional and cannot establish temporal ordering. Nevertheless, they are consistent with evidence that technology-enabled health transformation depends on leadership, human capability, infrastructure readiness, and organizational process alignment (3, 13, 14).

This pattern indicates that successful AI-driven digital health transformation depends not only on formal institutional capacity and readiness, but also on the operational mechanisms through which those capacities are translated into practice. Relevant health-systems and AI-governance literature similarly emphasizes leadership commitment, organizational capability, workforce competence, accountability, and infrastructure as enabling conditions for responsible technology-enabled performance (9, 17, 24, 27).

The subgroup comparisons indicated meaningful disparities by institution type and geographic region, with university hospitals and metropolitan institutions reporting higher readiness levels than MoHP and rural/Upper Egypt facilities. Because formal measurement invariance and path-comparison tests were not conducted, these results are interpreted descriptively. They nonetheless highlight a possible digital divide that future research should test more rigorously.

### 4.2 Comparison with existing literature

The findings align with literature showing that digital health transformation in low- and middle-income countries is often constrained by infrastructure deficits, workforce limitations, and fragmented governance (4, 5, 15). The Egyptian case adds evidence that these barriers are not isolated technical problems but mutually reinforcing institutional, administrative, and regulatory conditions.

The qualitative evidence also supports international AI-governance literature: participants identified regulatory uncertainty, data privacy, patient safety, bias, and accountability as barriers to responsible AI adoption. These concerns are consistent with recent AI-governance frameworks and maturity-model approaches for healthcare organizations (9, 10, 12, 26, 36).

Evidence from health-focused digital transformation research suggests that performance gains are strongest when digital capabilities are embedded in governance, trust, workforce capability, and performance-management routines rather than treated as isolated technical tools (3, 14, 37). These findings support the present study's emphasis on governance maturity, leadership support, IT infrastructure, and workforce skills as interdependent determinants of AI-driven public healthcare transformation.

### 4.3 Policy implications

The findings suggest several policy priorities for Egyptian health system actors. First, a national AI-in-health governance body could coordinate digital health policy across ministries, set standards for procurement and deployment, and clarify accountability for AI-enabled health systems. Such a body should include government, academia, industry, healthcare professionals, and patient/community representatives.

Second, the health inequities need to be addressed and investments in IT infrastructure need to be made systematically, taking into account the rural and underserved populations. This demands not only hardware and connectivity investments, but also the establishment of systems that integrate data across organizational and geographic areas. Addressing the infrastructure gaps identified in this study will be crucial in achieving the goals of the National Digital Health Strategy of Egypt 2025–2029.

Third, a National Digital Health Workforce Strategy is needed to address documented gaps in digital literacy and health-IT skills. This approach should involve reform of pre-service healthcare education, systematic in-service training, and development of career pathways for health-informatics professionals. The World Health Organization's Global Strategy on Digital Health provides a useful policy framework for this agenda (38).

Fourth, the adoption of AI specific health laws should be expedited, expanding on the current Personal Data Protection Law with a focus on algorithmic transparency, clinical validation, liability, and ethical guidelines for the use of AI in health-care. These regulations should be formulated in an inclusive, multi-stakeholder process including healthcare providers, technology developers, patient representatives and ethicists.

### 4.4 Limitations

Several limitations should be noted. First, the cross-sectional design prevents causal inference; therefore, all SEM paths and indirect pathways are interpreted as associations and hypothesis-generating mechanisms. Second, the study relied on self-reported survey data, which may be affected by common method bias and social desirability, although anonymity, neutral item wording, construct-block separation, and CFA-based sensitivity checks were used to reduce this risk. Third, the sampling frame was built from participating institutions rather than from a complete national registry of all eligible healthcare administrators, limiting the ability to claim national representativeness. Fourth, subgroup comparisons by institution type and region were exploratory and were not based on formal measurement invariance testing. Fifth, private healthcare institutions were not included, although they are increasingly important in Egypt's health system. Sixth, the study measured readiness and perceived transformation success rather than actual AI system performance, clinical outcomes, algorithmic accuracy, or post-deployment safety events. Finally, because AI technologies and regulatory expectations evolve rapidly, the findings should be updated through longitudinal and comparative research.

## 5 Conclusions

This study offers empirical evidence that institutional capacity, administrative readiness, organizational governance, leadership support, IT infrastructure, staff training and skills, and regulatory frameworks are closely associated with perceived AI-enabled digital health transformation success in Egypt. The model explained 68% of the variance in DHTS, while the qualitative strand clarified how fragmented governance, infrastructure gaps, workforce limitations, regulatory ambiguity, and resource constraints shape readiness in practice.

The National Digital Health Strategy 2025–2029 represents an important policy opportunity, but implementation will require institutional strengthening, governance coordination, AI-specific safeguards, infrastructure equity, and workforce development. Without these foundations, digital and AI-enabled technologies may fail to deliver their expected public value and may widen existing health system inequities.

Future studies should use longitudinal designs, national sampling frames, direct indicators of AI governance maturity, and objective measures of digital health performance. Comparative studies across MENA countries and in-depth case studies of successful Egyptian digital health implementations would also help identify transferable lessons and scalable governance models.

## Data Availability

The raw data supporting the conclusions of this article will be made available by the authors, without undue reservation.
